# InsertionMapper: a pipeline tool for the identification of targeted sequences from multidimensional high throughput sequencing data

**DOI:** 10.1186/1471-2164-14-679

**Published:** 2013-10-04

**Authors:** Wenwei Xiong, Limei He, Yubin Li, Hugo K Dooner, Chunguang Du

**Affiliations:** 1 Department of Biology and Molecular Biology, Montclair State University, Montclair, NJ 07043, USA; 2 Waksman Institute, Rutgers University, Piscataway, NJ 08854, USA

**Keywords:** Next-generation sequencing, Sequence identification, Target enrichment, Multidimensional pooling

## Abstract

**Background:**

The advent of next-generation high-throughput technologies has revolutionized whole genome sequencing, yet some experiments require sequencing only of targeted regions of the genome from a very large number of samples. These regions can be amplified by PCR and sequenced by next-generation methods using a multidimensional pooling strategy. However, there is at present no available generalized tool for the computational analysis of target-enriched NGS data from multidimensional pools.

**Results:**

Here we present InsertionMapper, a pipeline tool for the identification of targeted sequences from multidimensional high throughput sequencing data. *InsertionMapper* consists of four independently working modules: Data Preprocessing, Database Modeling, Dimension Deconvolution and Element Mapping. We illustrate InsertionMapper with an example from our project 'New reverse genetics resources for maize’, which aims to sequence-index a collection of 15,000 independent insertion sites of the transposon *Ds* in maize. Identified sequences are validated by PCR assays. This pipeline tool is applicable to similar scenarios requiring analysis of the tremendous output of short reads produced in NGS sequencing experiments of targeted genome sequences.

**Conclusions:**

InsertionMapper is proven efficacious for the identification of target-enriched sequences from multidimensional high throughput sequencing data. With adjustable parameters and experiment configurations, this tool can save great computational effort to biologists interested in identifying their sequences of interest within the huge output of modern DNA sequencers. InsertionMapper is freely accessible at https://sourceforge.net/p/insertionmapper and http://bo.csam.montclair.edu/du/insertionmapper.

## Background

Next-generation sequencing (NGS) technology facilitates genome sequencing in an unprecedented high throughput and cost-effective way [1]. Since the entire genome sequencing of major model organisms, huge numbers of short reads have been generated at a relatively low cost compared to prior Sanger sequencing technology [2]. Although the cost per read has been decreasing dramatically in recent years [3], a great effort is still required to assemble billions of whole genome short reads in the absence of a reference genome.

Alternatively, NGS sequencers can be used to sequence targeted genomic regions from a very large number of samples arranged in multidimensional pools. Multi-dimensional pooling, specifically 3-D pooling, has been used to identify a specific individual within a group of up to several thousand individuals with a limited number of PCRs [4]. The same concept applies to target-enriched high throughput sequencing libraries. Genomic regions are sheared to a specific size range, selectively amplified and then sequenced. Experimental design varies depending on the target, yet most involve nested PCR amplification in order to increase the representation of the desired DNA sequence segments. Different protocols have been developed to achieve the best balance between sensitivity, specificity and uniformity [5].

Short-read sequencers can yield tens of millions of reads in a single run, allowing many targets to be sequenced simultaneously as long as the coverage is deep enough for analysis. In mutation discovery, hundreds to thousands of mutants may need to be tested in parallel. A protocol for multiplexed barcoded sample pooling has been developed [6]. Samples are distinguished by their locations in the multidimensional sample pool, each pool barcoded with a unique short DNA sequence. After sequencing, reads can be deconvoluted according to barcodes and coordinates in the sample pool. In an appropriate experimental design, thousands of samples can be sequenced in one run. However, there is at present no available generalized tool for the computational analysis of target-enriched NGS data from multidimensional pools.

Here we present InsertionMapper, a pipeline tool for the identification of targeted sequences from multidimensional high throughput sequencing data. From the design of primer for different target elements to the arrangement of samples in pools, all parameters are adjustable for specific experiments. Identified elements are categorized into two grades, each with an empirical filter threshold. InsertionMapper consists of four independently working modules: Data Preprocessing, Database Modeling, Dimension Deconvolution and Element Mapping. We illustrate the use of InsertionMapper with an example from our maize genome project that aims to create a large set of single gene knockouts with a transgenic *Ds* transposon marked with green fluorescent protein (*Dsg*). This pipeline tool is applicable to similar situations that require analysis of the tremendous output of short reads produced in NGS sequencing experiments of targeted genome sequences.

## Implementation

### Modules overview

**Figure 1:**
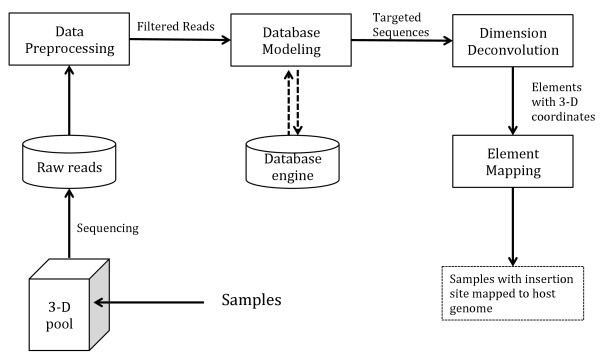
Overview of InsertionMapper modules.

InsertionMapper consists of four modules, each designed for a single function or a group of cohesive functions in the whole workflow (Figure 1). The output from one module sequentially serves as the input for the next module downstream in the pipeline. Each module works independently as a stand-alone command-line program, with configurable settings for contingencies of data quality, experimental design and primers. While modules are invoked over a system terminal, tasks can be easily organized by calling them from system shells (e.g. bash script for Unix-based operating system, and batch file for Windows) for higher efficiency.

Modules will be illustrated for the most common scenarios of 3-D pooling [7,8] with the three dimensions named “plate”, “row” and “column”, though InsertionMapper works for even higher dimensions if designed accordingly. One sample is placed in a single unit container (a “well”) in the sample pool denoted by its 3-D coordinates. There is one enriched targeted sequence in each of our samples, namely the *Dsg* insertion site to be identified by this InsertionMapper tool.

### Data preprocessing

This module takes high throughput reads from the DNA sequencer as input, converts reads format when necessary, and filters out unrelated reads based on experiment design (e.g. primer sequence). Because of sequencing error, mispriming, and/or the amplification of related endogenous elements in the host genome (see, e.g., endogenous *Ds* elements in maize genome in reference [9]), raw reads have to be filtered, otherwise the undesired reads would hinder further analysis by diminishing the significance of desired ones. Ideally, the output of the Data Preprocessing module retains only the reads of the target-enriched regions and passes them to the next module. Regular expression is adopted for customized filtering requirements, for instance, allowing read mismatches to tolerate minor sequencing errors, extending match length for extra 'identifier sequence’ to exclude misprimed reads, and preloading endogenous elements from a configuration file to avoid them. Furthermore, endogenous elements can be also inferred in the Dimension Deconvolution module.

Next-generation sequencers, such as SOLiD and Illumina, support the FASTQ format for raw reads, a text-based format for nucleotide sequences and their quality scores, whereas the FASTA format, which includes only sequence composition and annotations, is in more general use because of its simplicity. Therefore, in this module, data conversion from FASTQ to FASTA is provided as an option, in case users plan to analyze raw sequences with other tools.

Filter criteria comprise barcodes of libraries, primers, and a short piece of 'identifier sequence’. Any reads that do not match certain criteria are removed. Read prefixes (e.g. primers) are trimmed off in the output files since they do not provide information after filtering. For DNA transposons, target site duplications (TSD) are sliced from full-length reads as index sequences for database operations in the following module.

### Database modeling

This module imports tabular text files of filtered reads from the Data Preprocessing module into a relational database. If there are *N* types of targeted sequences in all *L* libraries, the total count of data tables will be *N* * *L*. Every data table shares the same structure, with fields including sequence index (e.g. TSD for DNA transposons), full-length read and raw read ID.

Without a powerful database engine, even filtered reads can be overwhelming (usually up to Gigabytes) for the computer memory to handle. A typical Database Management System (DBMS) allows users to add, delete, access, modify, and manipulate massive data very efficiently in terms of memory footprint and CPU usage. Especially when reads have to be grouped, sorted, and ranked for every type of targeted sequences in all dimensions, using sequence indices as supported by DBMS will save significant computation resources.

InsertionMapper eliminates the need for the user to write SQL queries by embedding query logic and generating SQL sentences on the fly, exchanging data through the JDBC API from the standard Java library (JRE version 1.6+). Considering that users may run InsertionMapper on different platforms, an SQL connection string could be configured by users with sufficient privilege to make changes to the database.

### Dimension deconvolution

The Dimension Deconvolution module is the heart of InsertionMapper. Robustness is crucial to the success of deciphering sample coordinates from multidimensional pools since data quality may vary greatly and reads retained through the Data Preprocessing module can still contain variable noise. The premise of this module is that the chance of a sequence being amplified and sequenced at deep coverage is much higher for the targeted sequences than for undesired noise sequences; on the other hand, any endogenous elements (rare with transgene-based PCR primers) are inferred by their high coverage in more than 50% of all data pools due to abundance from the host genome background, in contrast to real elements prominent in only 3 data pools. Once inferred, any endogenous elements can be saved as known ones into a configuration file for future filtering out of undesired reads at the beginning.

Read coverage is defined as the number of reads sharing the same sequence indices in one library and is normalized according to the total number of reads in that library in order to reduce bias from sample preparation or sequencing. Reads are grouped by their sequence indices to get normalized coverage, and then sorted by coverage in a descending order. Thereafter, reads are ranked sequentially from one to the total number of sequence types in the library.

Based on the following criteria, deconvoluted targeted sequences are categorized into two grades:

Grade-1: For a 3-D pool comprising *P* plates, *R* rows and *C* columns, there are *R* × *C*, *P* × *C* and *P* × *R* genuine elements in each plate, row and column respectively. Genuine elements ideally exist with high coverage only in their true *P*, *R*, and *C* dimensions, so the top *R* × *C*, *P* × *C* and *P* × *R* ranking elements in the respective dimensions are taken to be the genuine ones. In practice, we introduce a threshold *TH_G1* (default value 1.5, adjustable with varying reads quality) to allow more candidates for screen. In plate, row and column pools, the top *TH_G1* × *R* × *C*, *TH_G1* × *P* × *C* and *TH_G1* × *P* × *R* ranking elements are picked out as Grade-1 candidates. If a candidate exists in one and only one unique combination of the three dimensions, it is chosen as a Grade-1 targeted sequence. In rare occasions, candidate elements can be assigned to multiple wells as per their ranks, which should also be recorded for further investigation (in our reverse genetic resource, these would correspond to premeiotic transpositions that get incorporated into more than one progeny and, therefore, more than one well).

Grade-2: Ideally, all wells in sample pools are occupied by genuine Grade-1 elements. In practice, some of them lack viable candidates because of stringent selection criteria, thus requiring the parameters to be loosened in order to identify additional candidates. One possibility is that genuine elements do not have high enough coverage in any pool in a particular dimension, but their coverage in one of the pools is still much higher than in the other pools. Therefore, another threshold *TH_G2* (default value 3) is introduced to include elements with two “good” dimensions, as required by Grade-1, and one “not-so-good” dimension, in which elements are beyond Grade-1 range, but high enough to meet the Grade-2 threshold.

This module dynamically generates SQL queries for all wells, retrieves data from DBMS, and applies user-defined criteria to obtain targeted sequences in two grades. Ambiguous and suspicious well assignments are marked for further investigation and verification. A set of HTML pages is generated to present visual summaries of well assignment, along with a FASTA format file containing all targeted sequences with their well locations for the next Element Mapping module.

### Element mapping

Targeted sequences are mapped to the host genome (generally, the single reference genome available) based on sequence similarity. A sequence alignment module is not implemented here. Instead, the Element Mapping module takes output from known alignment software (BLAST and BOWTIE2 are supported) and compares the matched locations to user-chosen genome annotation files.

If targeted sequences come from the same line as the genome used for mapping, all should be mapped to at least one locus. Otherwise, unmapped sequences are possibly due to genome structure variation [10] or to an incomplete reference genome.

Primers are normally designed for both 5’ and 3’ ends of the insertion element in order to target both sides of the insertion. The sequences generated from each end are filtered and treated independently based on end-specific primers. Being mapped to the same location on the host genome constitutes strong evidence that both terminal sequences are genuine.

## Results and discussion

The InsertionMapper pipeline tool was originally designed for the identification of *Ds-*targeted sequences from NGS data, but should suit most scenarios in which biologists want to identify and map targeted sequences from large multidimensional sample pools using high throughput sequencing technology. The experiment methods we used for *Ds*-targeted sequences are detailed in Additional file 1.

### Identification of *Ds*-targeted sequences

In our NSF-PGRP-funded project, we are creating a maize reverse genetics resource based on the classical *Ds* transposon [11]. The resource consists of a large sequence-indexed library of at least 15,000 transposants, i.e. lines carrying transposed *Ds* elements, most of which are inserted into a gene or its regulatory region [12,13]. These elements have transposed from single T-DNA platforms [14] scattered around the genome. Our transgenic *Dsg* (green *Ds*) element is marked with the jellyfish green fluorescence proteins (GFP), allowing amplification of *Dsg*-adjacent sequences with nested PCR primers based on GFP and the region at the end of *Ds*[15]. DNA samples of pooled transposants are sheared and amplified in multiple phases so as to enrich for *Dsg* elements and their adjacent insertion sites for high throughput NGS.

A 3-D pooling strategy [7] is adopted here to sequence large numbers of transposant samples efficiently. Each sample is placed into one of 96 wells (12 columns by 8 rows) on a particular plate, and plates are stacked up to accommodate a large number of samples, each in a 3-D well with unique coordinates. Each pool is barcoded with a short unique sequence before being fed into a SOLiD 5500 sequencer. In practice, the number of samples in each dimension has to be chosen judiciously so as to balance sequencing efficiency with read length after all prefixes to reads (including barcodes and primers) are trimmed off.

The raw data consist of reads assigned to different libraries according to their barcodes. In each run, 960 samples in a “cube” of 10 plates, 8 rows and 12 columns generate 30 libraries of raw reads from the sequencer.

**Figure 2:**
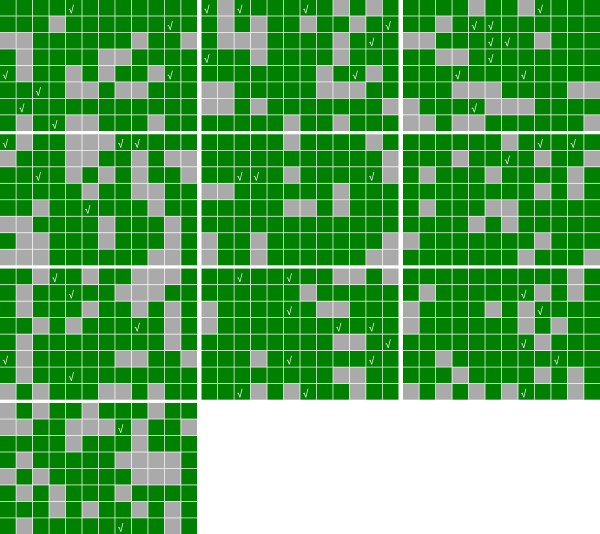
**Identified *Dsg *sequences among 960 samples.** Small squares, large rectangles, rows, and columns denote sample wells, plates, rows, and columns in the 3-D sample pool, respectively. Wells with identified elements are in green, while those without an assignment are in gray. Green squares with checkmarks are samples validated by PCR assay.

**Figure 3:**
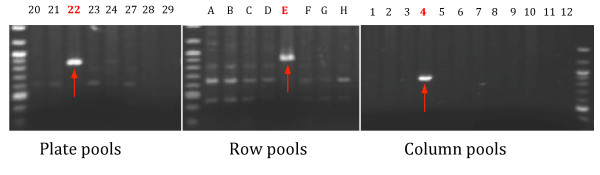
**PCR verification of the sample in well 22E04 (plate 22, row E and column 04).** Primers were designed according to the sequence assigned to well 22E04 by InsertionMapper, and tested in plate, row and column pools respectively. Plate numbers, row letters, and column numbers are labeled above the respective PCR product images. Clear bands (red arrows) are observed exclusively in the expected dimensions (colored in red), thus verifying the authenticity of the sequence identified in well 22E04.

As shown in Figure 2, out of 960 samples placed in wells, *Dsgs* from 728 wells were identified and 67% of them were anchored to unique loci of the maize B73 genome, the only public maize genome. A putative sequence is validated only if confirmed in all three dimensions, as shown in the example in Figure 3. Among the sample pools, only plate 22, row E, and column 4 give the expected PCR band (red arrows). Among unmapped *Dsg*s, some fell in repetitive regions and others did not map to the reference genome of the inbred B73, most probably because our genetic resource is in a W22/B73/A188 hybrid genetic background.

### *Dsg* transposition in the maize genome

Single-gene-knockout mutants caused by the insertion of transposable elements are of great utility to biologists. We annotated *Dsg*s that transposed into unique loci, based on gene annotations provided by MaizeGDB [16]. 444 *Dsg*s had inserted inside or within ±1 kb of genes. Many of these transpositions will result in loss-of-function mutations, which are powerful tools for biologists to analyze gene function. Table 1 exemplifies data for 10 wells from plate 20 and plate 21. Each *Dsg* is inserted in a unique location in the maize genome, more interestingly, in an exon of a gene, and has most likely knocked out the function of that gene. A full list of sequence-indexed *Dsg*s can be viewed at our project website http://acdsinsertions.org/showt/dsg.

**Table 1:** ***Dsg *transposition in maize genome**

**Well**	**Chr**	**Location**	***Dsg *insertion sequence**	**Gene model**	***Dsg *exon insertion**	**Gene description**
20G08	1	275382862	GTAGCATC…	GRMZM2G010034	2/6	Uncharacterized protein
20H04	2	40009418	CAGATGAC…	GRMZM2G327295	2/4	Putative copper transport protein
20H07	10	10196376	CAAGTGAA…	GRMZM2G046284	2/2	Fructose bisphosphate aldolase
20H11	9	154438893	GTCGCCGA…	GRMZM2G092718	3/3	Putative copper transport protein
21B12	9	152260129	GGCGTGCA…	GRMZM2G438840	2/2	Putative leucine rich-repeat transmemberane protein kinase
21B12	9	102784988	GTCTGAAC…	GRMZM2G006790	1/4	Putative calcium binding protein CML21
21C05	7	164656311	TCTGCGGC…	GRMZM2G082205	2/22	Putative protein
21D01	9	154438893	GTCGCCGA…	GRMZM2G092718	3/3	Putative copper transport protein
21D03	2	40150103	GTGCCATT…	GRMZM2G050435	3/5	Hypothetical protein
21D06	7	11443820	CTTGTATA…	GRMZM2G401308	5/5	Histone H1-3

10 *Dsg*s from plate 20 and plate 21 are shown with their 3-D coordinates in sample pools (**Well**), chromosome (**Chr**) locations on the maize B73 genome (**Location**), 8-bp target site duplication (TSD) of host junction sequences (***Dsg *insertion sequence**), sequences within ±1kb to the insertion site (**Gene model**), the exon where *Dsg* inserted relative to the total number of exons in that gene (***Dsg *exon insertion**) and the **gene description**. A full list of sequence-indexed *Dsg*s can be viewed at our project website http://acdsinsertions.org/showt/dsg.

### Scalability and performance

The main body of InsertionMapper is written in the Scala programming language, and runs on top of the java virtual machine (JRE 1.6+ required), which is compatible with all mainstream operating systems. In the Data Preprocessing module, large-scale raw reads are filtered one by one, where the massive data are not loaded into computer memory at once, causing no memory pressure at all to any modern computer. The filtered reads are then manipulated in DBMS that is highly optimized for a huge amount of data. Generally the InsertionMapper scales very well with raw reads up to tens of gigabytes.

In our last run of the *Dsg* experiment, the 19.2 GB sized raw reads generated by Illumina MiSeq sequencer comprise *Ds* elements from 10 plate pools, 12 column pools and 8 row pools. On our MacPro 4.1 (Mac OS X 10.5.8) with 2 quad-core Intel Xeon 2.26 GHz processors and an 8 GB memory, it takes about 27 minutes to identify all the target-enriched elements from the huge amount of raw reads. The MySQL Community Server 5.1.39 is used as DBMS.

## Conclusions

InsertionMapper is designed for the identification of targeted sequences from multidimensional high throughput sequencing data, and is proven efficacious for the identification of target-enriched Dsg elements in our project. PCR assays were conducted to validate the identified sequences. With adjustable parameters and experiment configurations, InsertionMapper can save great computational effort to biologists interested in identifying their sequences of interest within the huge output of modern DNA sequencers.

## Availability and requirements

**Project name:** InsertionMapper

**Project home page:**https://sourceforge.net/p/insertionmapperorhttp://bo.csam.montclair.edu/du/insertionmapper.

**Operating systems:** Mac OS X, Windows, and Unix-like operating systems that support java virtual machine version 1.6 or above.

**Programming language:** The main body of InsertionMapper is written in Scala 2.10, while SQL scripts are used in the configuration file for database manipulation.

**License:** GNU

**Any restrictions to use by non-academics:** No

## Competing interests

The authors declare that they have no competing interests.

## Authors’ contributions

Designed and wrote the software: WX CD. Created *Dsg* launching platforms: YL HKD. Performed the PCR validation experiments: LH. Analyzed the data: WX LH HKD CD. Wrote the paper: WX HKD CD. All authors read and approved the final manuscript.

## Supplementary Material

Additional file 1 Experiments Methods. Click here for file
