# Supplementary material for: InsertionMapper: a pipeline tool for the identification of targeted sequences from multidimensional high throughput sequencing data
Source: BMC Genomics. 2013 Oct 4;14:679. doi: 10.1186/1471-2164-14-679 (PMC3850689; doi:10.1186/1471-2164-14-679)
Supplement: Additional file 1 — Experiments Methods. [file 1471-2164-14-679-S1.docx]

**Experiment Methods**

The 960 transposants from each platform were assembled into 10 x 8 x 12 “cubes” consisting of 10 pools of 96 individuals (“plates”), 8 pools of 120 individuals (‘rows”), and 12 pools of 80 individuals (“columns”). Thus, 30 sequencing libraries are needed for each “cube” of 960 individuals. Every transposant contributes DNA to 3 pools, so its *dsg* sequence presented in high coverage in 3 of the 30 sequencing libraries. The intersection of the 3 dimensions (e.g., plate 22, row E, column 04) identifies the address of the individual transposant and a BLAST search of the maize genome maps the *dsg* sequence to a location in one of the 10 maize pseudomolecules.

NGS libraries require very high quality DNA, which we prepared as follows. A 96-­‐

well-­‐plate format is mimicked by assembling four 24-­‐cell nursery trays next to each other. Every other day, 8 trays (2 plates) were planted with 5 seed from each transposant per cell. After 2 weeks, leaves from 3 seedlings in each cell were sampled and pooled, as described below. The fourth seedling was sampled separately into a 96-­‐well plate and stored at -­‐80°C for later PCR verification. A roughly equal amount of leaf tissue from each line was placed into three 50-­‐ml centrifuge tubes corresponding to a plate, a row, and a column pool. Each plate contributed 21 tubes of pooled tissue: 1 plate tube, 8 row tubes and 12 column tubes. After sampling all 10 plates, the leaf tissues of same-­‐lettered row tubes and same-­‐numbered column tubes from different plates were pooled together to become the corresponding row and column pools of a 10x8x12 “cube”. The 30 pooled leaf tissues of the 960 transposants in each “cube” were ground to a fine

powder in liquid nitrogen. A large-­‐scale urea buffer method was used for DNA

extraction.

To produce a SOLiD sequencing library, we sheared pooled genomic DNA using a Covaris S2 single tube sample shearing system to avoid cross contamination. Sheared DNA was purified with AmPure XP beads to further reduce polysaccharide and polyphenol contaminants. The purified DNA was end-­‐repaired and ligated to splinkerette adaptors. Splinkerette-­‐PCR [[1](#_ENREF_1)] has proved very efficient for the isolation of transposon insertion sites [[2](#_ENREF_2)]. Its design ensures that the DNA cannot be amplified until synthesis initiates from a primer that binds to the target

sequence, e.g. *Ds**, greatly improving specific amplification of the desired sequences.

After PCR amplification with primers based on the splinkerette adaptor and the unique *GFP* sequence in *Ds**, excess adaptor and primer were removed. Molecules that have incorporated the biotinylated splinkerette primer were then captured with Streptavidin-­‐coated magnetic particles. We then amplified the bead-­‐bound library with nested PCR primers based on the splinkerette adaptor and the *Ds* end sequences. The nested PCR product was size-­‐selected in 2% metaphor gels for fragments 100 to 250-­‐bp long and provided to the Waksman Institute Genomics Facility for sequencing by SOLiD 5500xl technology.

**References**

1. Devon RS, Porteous DJ, Brookes AJ: **Splinkerettes--improved vectorettes for greater efficiency in PCR walking**. *Nucleic Acids Res* 1995, **23**(9):1644-1645.

2. Uren AG, Mikkers H, Kool J, van der Weyden L, Lund AH, Wilson CH, Rance R, Jonkers J, van Lohuizen M, Berns A *et al*: **A high-throughput splinkerette-PCR method for the isolation and sequencing of retroviral insertion sites**. *Nature protocols* 2009, **4**(5):789-798.
